# TMEM219 regulates the transcription factor expression and proliferation of beta cells

**DOI:** 10.3389/fendo.2024.1306127

**Published:** 2024-01-22

**Authors:** Francesca D’Addio, Emma Assi, Anna Maestroni, Giada Rossi, Vera Usuelli, Adriana Petrazzuolo, Marta Nardini, Cristian Loretelli, Moufida Ben Nasr, Paolo Fiorina

**Affiliations:** ^1^ International Center for Type 1 Diabetes (T1D), Pediatric Clinical Research Center Romeo ed Enrica Invernizzi, Department of Biomedical and Clinical Sciences (DIBIC), Università di Milano, Milan, Italy; ^2^ Division of Endocrinology, ASST Fatebenefratelli-Sacco, Milan, Italy; ^3^ Nephrology Division, Boston Children’s Hospital and Transplantation Research Center, Brigham and Women’s Hospital, Harvard Medical School, Boston, MA, United States

**Keywords:** type 1 diabetes, endocrine progenitors, TMEM219, IGFBP3, islets, mature and immature beta cells

## Abstract

Pancreatic beta cells replenishment is considered the next therapeutic option for type 1 diabetes; while stimulating endogenous beta cells proliferation is the “holy grail” for those patients with exhausted beta cell mass. Here we are demonstrating that the pro-apoptotic receptor TMEM219 is expressed in fetal pancreas, in beta cell precursors and in *in vitro* embryonic-derived endocrine progenitors. TMEM219 signaling negatively regulates beta cells at early stages and induces Caspase 8-mediated cell death. Pharmacological blockade of TMEM219 further rescued beta cell precursor and proliferation markers, and decreased cell death, both in islets and in *in vitro*-derived endocrine progenitors, allowing for beta cell preservation. While addressing the upstream controlling TMEM219 expression, we determined the TMEM219 miRNet; indeed, one of those miRNAs, miR-129-2, is highly expressed in human islets, particularly in patients at risk or with established type 1 diabetes. miR-129-2 mimic downregulated TMEM219 expression in islets, in *in vitro* embryonic-derived endocrine progenitors and in highly proliferating insulinoma-derived cells. Moreover, miR-129-2 inhibitor induced a TMEM219 overexpression in insulinoma-derived cells, which restored cell proliferation and functional markers, thus acting as endogenous regulator of TMEM219 expression. The TMEM219 upstream regulator miR129-2 controls the fate of beta cell precursors and may unleash their regenerative potentials to replenish beta cells in type 1 diabetes.

## Introduction

The existence of an islet neogenic niche, in which endocrine precursors may reside and enter into a process of differentiation in response to a selected stimulus/trigger, may represent a natural source of beta cells that can replenish those attacked and destroyed during the autoimmune response, as in type 1 diabetes (T1D) (1–3). No options are currently available to modulate islet neogenic niche and to take advantage of endocrine precursors in order to replenish the fading beta cell mass typical of T1D. The success of the latest clinical trials in which treatment compounds (e.g., verapamil), including immunosuppressants (i.e., teplizumab, abatacept), were tested in the earlier stages of the disease, pointed out at the benefit of having a beta cell “expander” (4–6). Interestingly, residual beta cells are detectable in nearly 20-30% of patients at 1 year from T1D diagnosis and may be detected in almost 10% of patients up to 40 years from the disease onset (7–9). This is, indeed, associated with an improvement in glycometabolic and clinical outcomes, and highlights the relevance of preserving a residual beta cell mass in patients with T1D (10). Therefore, the “holy grail” of regenerative approach in diabetes will be to stimulate the regeneration of endocrine precursors. We have recently reported that blockade of the TMEM219-mediated death signaling in insulin-expressing beta cells prevents their apoptosis and preserves both beta cell mass and function (11, 12). While in pathological conditions such as type 1 diabetes, the TMEM219 signaling is abnormally activated and it highly contributes to beta cell loss and dysfunction (11), in physiological conditions, TMEM219 acts mainly as a death receptor that regulates cells homeostasis and lifecycle. Indeed, by binding its circulating ligand Insulin-like growth factor binding protein 3 (IGFBP3), TMEM219 signals into target cells and induces a Caspase 8-mediated apoptosis (12, 13). TMEM219 has been also observed to activate cell autophagy *in vitro*, which also supports its role as a physiological regulator of cell death and survival (14). In this study, we will demonstrate that TMEM219 is expressed in beta cell precursors and in *in vitro*-derived endocrine progenitors and that targeting TMEM219 pharmacologically or inhibiting the upstream TMEM219 miRNet may unleash regenerative potentials of immature beta cells within the islets, which further facilitates the beta cells replenishment.

## Research design and methods

A detailed description of methods is reported in **Supplementary Information**.

### TMEM219 protein expression studies (immunostaining, immunofluorescence, ELISA)

Commercially available human fetal tissue sections (BE01015, US Biomax Inc, Derwood, MD, US) were immunohistochemically stained with rabbit polyclonal TMEM219 antibody (1:100, Sigma HPA059185). Immunofluorescence islets samples were examined using a confocal system (TCS SP2 Laser Scanning Confocal, Leica, Wetzlar, Germany) in multitrack mode with rabbit polyclonal TMEM219, guinea pig polyclonal insulin (1:100, DAKO, A0564), Guinea Pig polyclonal PDX1 (1:100, Abcam, Cambridge, MA 47308), mouse monoclonal aldehyde dehydrogenase (clone 44/ALDH, 1:1000 dilution, BD Transduction Laboratories, Franklin Lakes, NJ, USA) as primary antibodies. As secondary antibodies donkey anti-rabbit/donkey anti-mouse FITC, donkey anti-guinea pig TRITC and donkey anti-rabbit TRITC, (1:200, Jackson ImmunoResearch, West Grove, PA) were used. Nuclei were stained with DAPI (D-9542, Sigma), (**Supplementary Figures 1A1–A3**). As positive controls for PDX1 and ALDH immunofluorescence staining, the following cell lines were used (**Supplementary Figure 1B**): for PDX1, a human beta cell line (Betalox-5, **Supplementary Figure 1B1**), for ALDH a breast cancer cell line (MCF7, **Supplementary Figures 1B2, B3**). As negative controls (**Supplementary Figure 1C**), a colon adenoma cell line (CaCo2) was used for both PDX1 and ALDH staining (**Supplementary Figures 1C1, C2**). For TMEM219 immunofluorescence staining, we used CaCo2 as positive control (**Supplementary Figures 1C1, C2**) and a podocyte cell line (h-podo) as negative control (**Supplementary Figure 1C3**). For insulin a beta cell line was used as positive control (**Supplementary Figure 1B4**). TMEM219 expression was quantified by ELISA in lysates of differentiated h-ESCs and in rat INS-1 cells (MyBioSource ELISA, MBS9341285 and MBS9354074 respectively, San Diego, CA).

### Embryonic stem cell *in vitro* studies

Undifferentiated human embryonic stem cells (h-ESCs) were a generous gift of Prof. Thorsten Schlaeger’s lab and were differentiated by seeding 2.6 x 10^5^ cells/cm^2^ and using the STEMdiff™ Pancreatic Progenitor Kit (05120, STEMCELL Technologies, Vancouver, BC) from stage 0 to stage 4 according to the manufacturer’s instructions. Differentiation efficiency for h-ESCs usually achieves more than 60% of PDX1-expressing cells at stage 3 and up to 90% at stage 4 (15). Differentiation to stage 5 was performed as previously described (16, 17). Cell death was quantified by ELISA (Roche Diagnostics GmbH, 11544675001, Mannheim, Germany).

### *In vitro* studies

Human islets (11, 18, 19)/endocrine progenitors/INS-1 cells were cultured with/without IGFBP3 (R&D Systems, 8874-B3, 50 ng/ml), with/without ecto-TMEM219 (130 ng/ml in a 1:1 molar ratio, Genscript, Piscataway, NJ), generated based on the extracellular portion of TMEM219 (11), for 72 hours and then were used for mechanistic analyses. *In vitro* experiments also included transfection of human islets/endocrine progenitors, which were incubated with 37.5 ng of miRNa-129-2 mimic (HSA-MIR-129-5P, 339173YM00470931-ADB) or of miRNA-129 inhibitor (HSA-MIR-129-5P, 339121YI04102970-ADC), both from Qiagen (Valencia, CA) and with 6 µl HiPerFect Transfection Reagent (Qiagen). As negative control miRNA mimic (339173YM00479902-ADB, Qiagen) and miRNA inhibitor Control (339126YI00199007-ADC, Qiagen) were used. Proliferation was assessed by a BrdU incorporation assay (QIA58, Merck), in which cells were seeded and transfected as described above with miRNa-129-2 mimic or its negative control, and BrdU incorporation was detected at Day 1 and at Day 2 from transfection following manufacturer’s instructions.

### miRNA sequencing and miRNA detection by qRT-PCR

miRNA sequencing data were obtained from an Affymetrix Human Gene 2.0 ST array analysis previously performed on laser-captured islets of healthy subjects, T1D and T2D patients and AutoAb^+^ patients provided by Prof. Ivan Gerling (20). Data were expressed as fold change as compared to gene expression of healthy subject samples (11). The mirVana miRNA isolation kit was used to confirm miRNA expression and values were normalized using U6 small nuclear RNA as already described (21).

### Statistical analysis

Continuous variables are presented as means with standard errors and compared by using independent sample t-tests (Student t test, Mann Whitney test). For multiple comparisons, one-way ANOVA followed by Sidak/Holm-Sidak *post hoc* were used. Two-tailed P values of less than 0.05 were considered statistically significant.

## Results

### TMEM219 is expressed in beta cell precursors and in *in vitro*-derived endocrine progenitors

**Figure 1:**
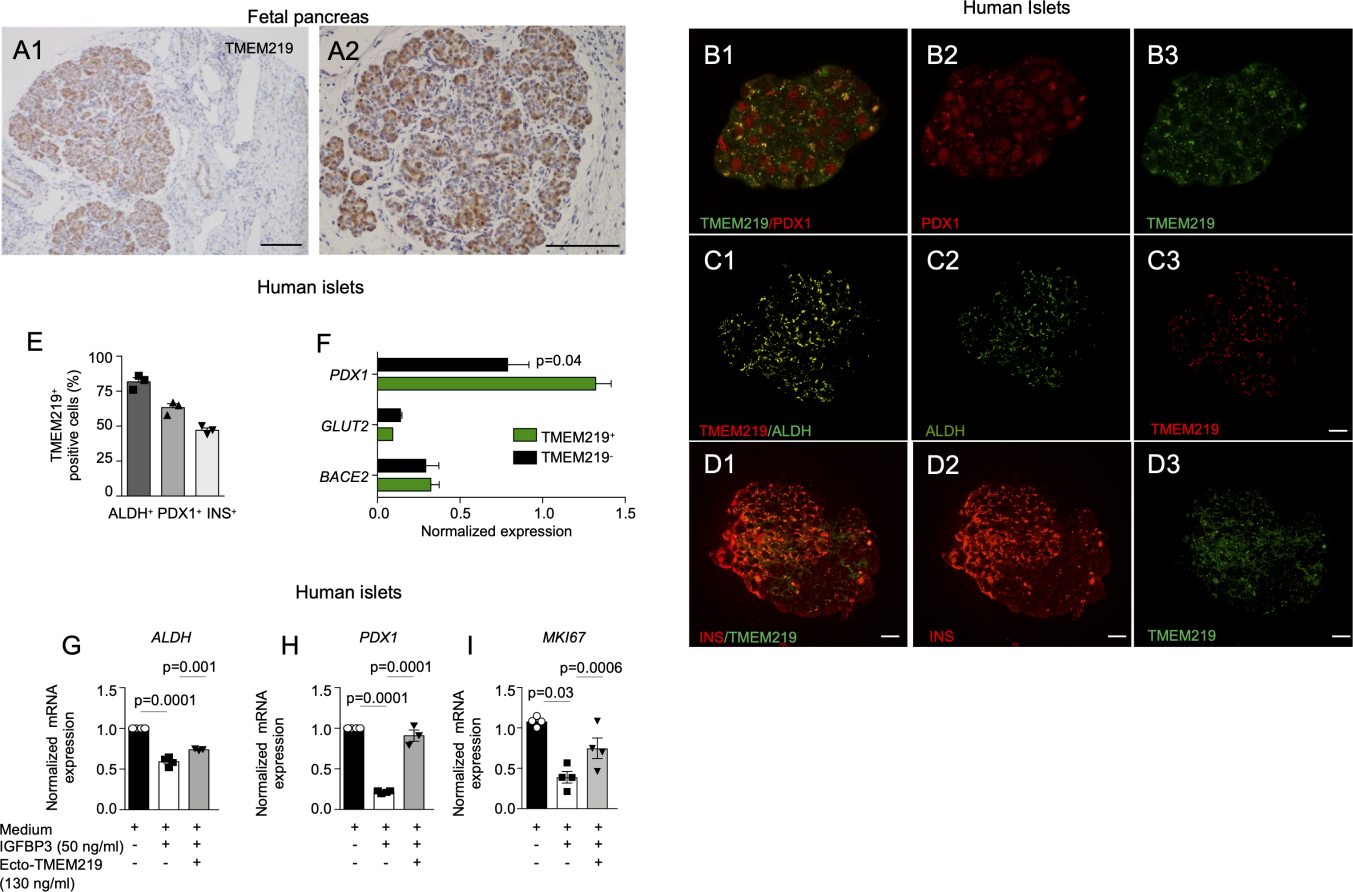
*TMEM219 is expressed in beta cell precursors.* **(A1-A2)** Representative images of TMEM219 immunostaining on fetal pancreata sections (at 5-months-old). Original magnification 10X **(A1)** and 20X **(A2)**, scale bar 300 and 150 μm respectively. **(B1-B3)** Representative pictures of PDX1^+^TMEM219^+^ (merge **B1)**, PDX1 (red, **B2)** and TMEM219 (green, **B3)** immunofluorescence in purified human islets of healthy donors (n=3). Original magnification 40X, scale bar 25 μm. **(C1-C3)** Representative pictures of ALDH^+^TMEM219^+^ (merge **C1)**, ALDH (green, **C2)** and TMEM219 (red, **C3**) immunofluorescence in purified human islets of healthy donors. Original magnification 40X, scale bar 25 μm. **(D1-D3)**. Anecdotical picture of TMEM219 (green, **D3**) and INS (red, **D2)** co-expression in purified human islets of healthy donors. Original magnification 40X, scale bar 25 μm. Merge is in **D1**. **(E)** Confocal analysis quantifying PDX1^+^TMEM219^+^, ALDH^+^TMEM219^+^ and INS^+^TMEM219^+^ cells in purified human islets of healthy donors (n=3). **(F)** Bar graph representing normalized expression of *PDX1*, *BACE2* and *GLUT-2* quantified by qRT-PCR in flow-sorted TMEM219^+^ and TMEM219^-^ cells obtained from dissociated human islets. **(G–I)** Bar graphs representing *ALDH* **(G)**, *PDX1* **(H)** and *MKI67* **(I)** mRNA expression analyzed by qRT-PCR in human islets cultured with/without IGFBP3 and in the presence or absence of ecto-TMEM219 (n=3-4). Data are expressed as mean ± standard error of the mean (SEM) unless otherwise reported. mRNA expression was normalized to *ACTB*. qRT-PCR, quantitative real-time polymerase chain reaction; PDX1, pancreatic and duodenal homeobox 1; ALDH, aldehyde dehydrogenase; BACE2, Beta-Secretase 2; GLUT-2, Glucose transporter 2; ecto-TMEM219, newly generated recombinant protein based on TMEM219 extracellular portion; SEM, standard error of mean.

To determine the role of TMEM219 signaling in beta cells at their early stage, we first demonstrated by immunostaining that TMEM219 is highly expressed in human pancreata at early stage of development (5-months-old) (**Figures 1A1, A2**). Next, we tested TMEM219 expression in purified human islets obtained from pancreata not suitable for donation (**Supplementary Table 1**) and observed at the confocal analysis substantial co-localization of TMEM219 with the precursor markers aldehyde dehydrogenase 1 (ALDH) and pancreatic and duodenal homeobox 1 (PDX1), (**Figures 1B1–B3, C1–C3, D1–D3, E**; **Supplementary Figures 1A1–A3, B, C**), thereby suggesting that TMEM219 may mark beta cell precursors within islets. This was further proved in flow-sorted TMEM219-positive cells obtained from dissociated human islets, in which the precursor marker *PDX1* was highly expressed as compared to the TMEM219-negative fraction (**Figure 1F**). We also obtained endocrine progenitors from *in vitro*-differentiated human embryonic stem cells (h-ESCs), (**Supplementary Figure 2A**), and demonstrated TMEM219 expression by qRT-PCR, targeted immunoassay and flow cytometry (**Supplementary Figures 2A–D**). Notably, TMEM219 protein was progressively less expressed in *in vitro*-differentiated h-ESCs at stages 4 and 3, and undetectable at earlier stages (1 and 2) (**Supplementary Figures 2B–D**). Our data demonstrate that *in vitro*-derived endocrine progenitors and beta cells in their early stages of maturation express TMEM219, which is then retained by islet mature beta cells (**Supplementary Figure 2E**).

### TMEM219 signaling controls beta cell precursors

Based on the observation that the pancreatic receptor TMEM219 is expressed in beta cell precursors, we next explored whether the TMEM219 signaling may act as an early-stage beta cell regulator. Interestingly, addition of the TMEM219 ligand IGFBP3, which activates TMEM219 signal into target cells, to purified pancreatic islets *in vitro* reduced mRNA expression of precursors markers *ALDH* and *PDX1* and of proliferation marker *MKI67*, which was next restored to normal in the presence of the TMEM219 inhibitor ecto-TMEM219 (**Figures 1G–I**). This suggests that the TMEM219 signaling may exert an effect on early-stage beta cells and that targeting the deleterious signal may be of benefit also for local endocrine precursors. To confirm this, we also cultured TMEM219-expressing h-ESC-derived endocrine progenitors in the presence of IGFBP3 and of the TMEM219 inhibitor ecto-TMEM219 and observed a decrease in apoptosis and a re-establishment of the TMEM219 downstream signaling through Caspase 8 (**Supplementary Figures 3A–C**). Besides, expression of precursor markers *PDX1* and *HNF6* (**Supplementary Figures 3D, E**) and that of proliferation marker *MKI67*, which decreased in the presence of IGFBP3, was also recovered (**Supplementary Figure 3F**). Our data demonstrate that beta cell precursors and h-ESC-derived endocrine progenitors suffer from a TMEM219-initiated Caspase 8-mediated damage.

### The TMEM219-related miRNet in human pancreatic islets

**Figure 2:**
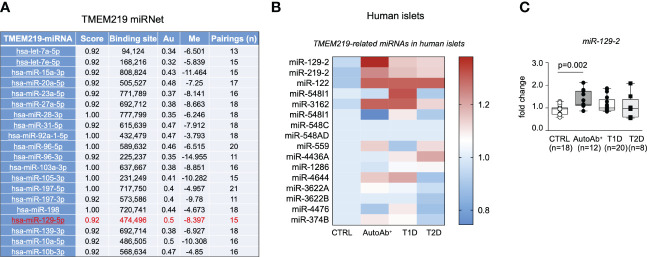
The TMEM219-related miRNet in human pancreatic islets. **(A)**. List of TMEM219-related miRNAs selected based on the mirwalk database scored higher than 0.92. Top 20 miRNAs are shown. In red the miR-129-2 is highlighted. **(B**, **C)**. Heat-map and box plots showing TMEM219-related miRNAs expressed in the miRNome of human laser-captured islets obtained from healthy controls, patients with T1D, T2D or at risk for T1D being positive for autoantibodies (mean value of expression were calculated for each sample). All box plots include the median line, the whiskers indicate the minimum and maximum value, and the box of the box plot illustrates the upper and lower quartile. CTRL, healthy volunteers; T1D, type 1 diabetes; pre-T1D, patients showing positive results for at least one autoantibody; AutoAb, autoantibodies; T2D, type 2 diabetes; qRT-PCR, quantitative real-time polymerase chain reaction.

In order to understand the upstream controlling TMEM219 expression, we generated a TMEM219-related miRNet, in which top 20 miRNAs interacting with the TMEM219 mRNA based on the miRWalk prediction analytic tool, have been included (**Figure 2A**). We next assessed the presence of those miRNAs, predicted to control TMEM219 expression, in human laser-captured pancreatic islets, thereby delineating the TMEM219-related islet miRNome (**Figure 2B**). In the analysis of miRNAs expression in laser-captured islets of heathy donors, of patients at risk for T1D (autoantibodies positive) or with established T1D, and of patients with type 2 diabetes (**Figure 2B**), miR-129-2 appeared highly expressed in islets of both patients at risk for T1D and with established T1D (**Figures 2B, C**), thus representing a relevant target that may modulate TMEM219 expression in pancreatic islets.

### miRNA mediated TMEM219 reduction unleashes proliferation of beta cells

**Figure 3:**
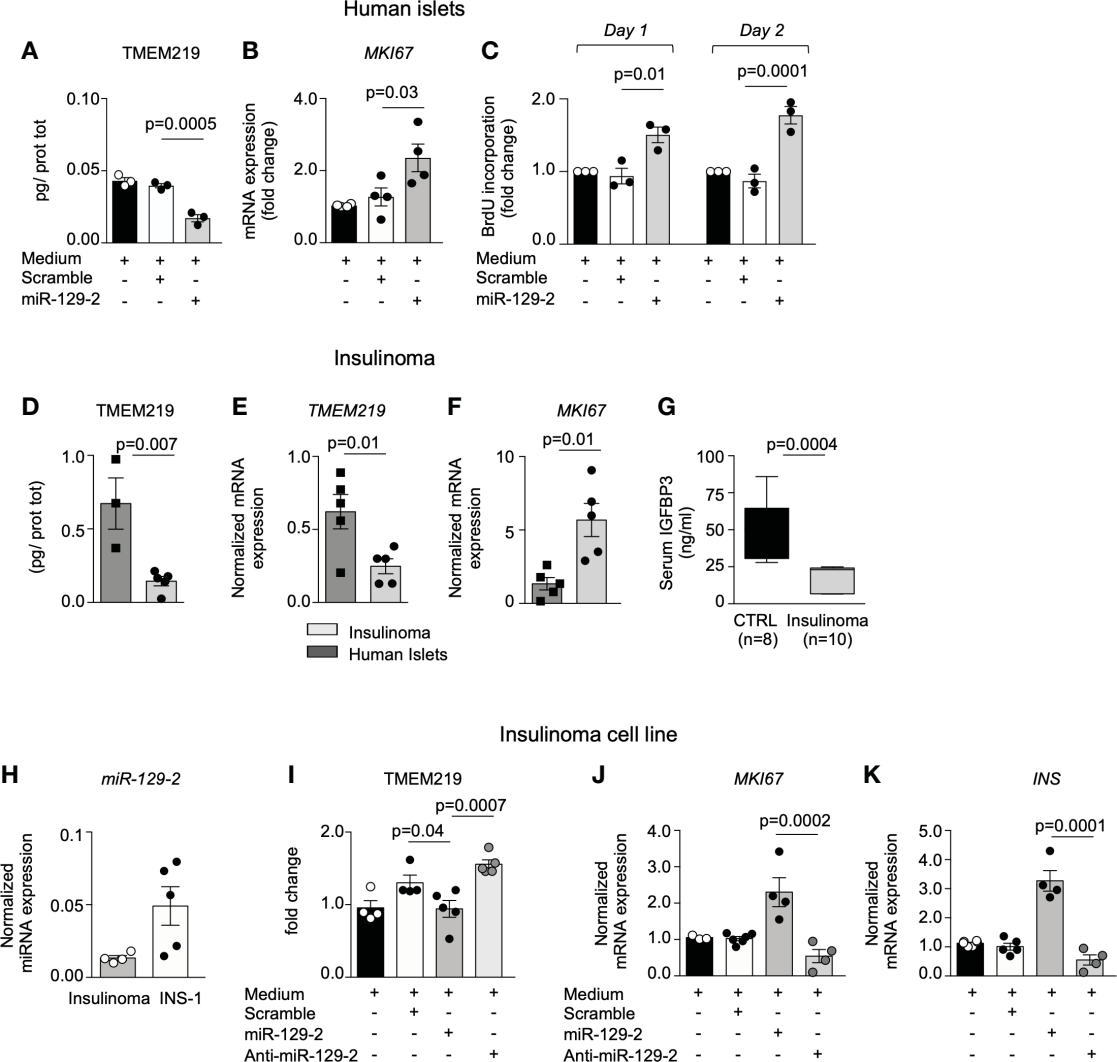
miRNA mediated TMEM219 reduction unleashes proliferation of beta cells. **(A)** TMEM219 protein quantification in human islets cultured with/without miR-129-2 mimic and its negative control (scramble), (n=3). **(B)** Bar graph representing *MKI67* mRNA relative expression in human islets cultured with/without miR-129-2 mimic and its negative control (scramble), (n=3). **(C)** Bar graphs quantifying proliferation in dissociated islet cells through BrdU incorporation at Day 1 and Day 2 after transfection with miR-129-2 mimic or its negative control (scramble), (n=3). Fold change was calculated on un-transfected cells. **(D**, **E)** Quantification of TMEM219 protein (ELISA) and mRNA (qRT-PCR) expression detected in excised insulinoma tissue (n=5) as compared to purified islets (n=3/4). **(F)** Bar graph representing M*KI67* mRNA relative expression in excised insulinoma tissue (n=5) as compared to purified islets (n=3/4). **(G)** Box plot showing peripheral IGFBP3 levels in patients with insulinoma (n=10, gray) as compared to healthy controls (n=8, black). **(H)** Bar graph representing *miR-129-2* relative expression (as compared to U6 miRNa) analyzed in insulinoma and in INS-1 cells (n=4/5). **(I)** Quantification of TMEM219 protein expression in INS-1 cells cultured with/without miR-129-2 mimic or with miR-129-2 inhibitor (anti-miR-129-2) (n=5). **(J**, **K)** Bar graph representing *MKI67* and *Insulin* mRNA relative expression in INS-1 cells cultured with/without miR-129-2 mimic or with miR-129-2 inhibitor (n=4). Experiments were performed in duplicates and at least three independent experiments were conducted. Data are expressed as mean ± standard error of the mean (SEM) unless otherwise reported. mRNA expression was normalized to *ACTB*. CTRL, healthy volunteers; INS, insulin; anti-miR-129-2, miR-129-2 inhibitor; MKI67, Ki67 mRNA marker; qRT-PCR, quantitative real-time polymerase chain reaction.

Based on the evidence that the TMEM219-related miR-129-2 is constitutively present in human pancreatic islets, we tested the miR-129-2 mimic through cell transfection in human islets and in *in vitro* differentiated endocrine progenitors (**Figures 3A–C**; **Supplementary Figures 3G–I**) and first confirmed that expression of TMEM219 was downregulated (**Figure 3A**; **Supplementary Figure 3G**). We also observed that the miRNA mediated TMEM219 reduction was associated with an increased proliferation rate in human islets (**Figure 3C**; **Supplementary Figure 3J**), higher mRNA expression of the proliferation marker *MKI67* (**Figure 3B**) and of *Insulin*, the later more evident in *in vitro* differentiated endocrine progenitors (**Supplementary Figures 3H, I**). To better understand the link between TMEM219 signal and proliferation of beta cell precursors, we took advantage of samples obtained from patients with insulinoma, in which pancreatic beta cells are prone-to-proliferate. A low expression of both TMEM219 mRNA and protein (**Figures 3D, E**) and a high mRNA expression of *MKI67* (**Figure 3F**), were evident in the insulinoma samples, with circulating levels of the TMEM219 ligand IGFBP3 also being reduced (**Figure 3G**), thus suggesting a dysregulation of the TMEM219 signaling. We next demonstrated that low expression of TMEM219 in insulinoma-derived cells was associated with detectable expression of the endogenous miR-129-2, which was also highly expressed in the insulinoma cell line INS-1, thereby confirming that TMEM219 expression is under the control of miR-129-2 also in prone-to-proliferate beta cells (**Figure 3H**). Interestingly, when testing miR-129-2 transfection in the insulinoma cell line, we confirmed a decrease in the expression of TMEM219 (**Figure 3I**), but more remarkably we detected an increase in *MKI67* and *Insulin* mRNA levels (**Figures 3J, K**). Finally, near-normalization of TMEM219 expression with a specific miR-129-2 inhibitor was associated with a decrease in mRNA levels of proliferation marker *MKI67* and *Insulin* (**Figures 3J, K**). Overall, our findings demonstrate the key role of TMEM219 expression in directing cell proliferation or death, and that controlling the TMEM219 signaling in beta cell precursors may unleash their regenerative and differentiation abilities to balance beta cell loss and damage such as in T1D.

## Discussion

In this study we demonstrated that the TMEM219 signaling is active in beta cells at their early stage of maturation and it may serve as a target to unleash regenerative potentials of precursor cells and help to replenish the beta cell mass in disease conditions such as T1D. The discovery of an islet neogenic niche in murine pancreatic islets, enriched in virgin beta cells, with an immature beta cell signature and unable to respond to glucose stimulation but positive to insulin staining (1, 2), already confirmed that progenitors of beta cells exist in murine islets and may serve to differentiate and generate new beta cells ultimately to restore the islet mass. Our study suggests that TMEM219 pathway is a key signaling that controls self-renewal abilities and proliferation of those beta cell precursors and that inhibition of TMEM219 may result in promoting the replenishment of beta cells. Indeed, low expression of TMEM219 was found in insulinoma-derived cells, in which beta cells are prone to proliferate, as well as low levels of the TMEM219 ligand IGFBP3 were detected in the circulation, thereby confirming a dysregulation of the TMEM219 signaling pathway when beta cell homeostasis is perturbed. This further suggests that controlling the TMEM219 signaling, with a TMEM219 inhibitor or with a miRNa mimic, may result in protecting beta cell precursors from damage and in promoting their renewal abilities. The fact that T1D is now viewed not only as a pure autoimmune disease, but rather as a condition, in which also the beta cells are more vulnerable, further suggests to directly target the exhausted beta cell mass (22, 23), which require an appropriate replacement. In this regard, several progresses have been reported in the stem cell-based generation of beta cells *ex vivo*, starting from induced pluripotent stem cells, now able to release insulin in a glucose-dependent manner (24–26). However, the “holy grail” of regenerative strategies in T1D will be to promote self-renewal, proliferation, and differentiation of endogenous pancreatic endocrine progenitors to generate new insulin-producing beta cells. Therefore, targeting pathways that controls the fate of beta cells at their early stage of maturation, such as the TMEM219 signaling, may ultimately allow us to unlock the potential of a local islet neogenic niche. Our strategy may unleash the endogenous regenerative abilities of the endocrine pancreas, ultimately to ease a clinically meaningful restoration of human beta cell mass.

## Data availability statement

The data presented in the study are deposited in the Dataverse repository, “TMEM219 pathway in beta cells”. DOI: https://doi.org/10.13130/RD_UNIMI/57PFIM.

## Ethics statement

The studies involving humans were approved by Ethic Committee Milan Area 1 and Niguarda Cà Granda Ethics Board, Milan, Italy. The studies were conducted in accordance with the local legislation and institutional requirements. The human samples used in this study were acquired from primarily isolated as part of a previous study for which ethical approval was obtained. Written informed consent for participation was not required from the participants or the participants’ legal guardians/next of kin in accordance with the national legislation and institutional requirements.

## Author contributions

FD’A: Conceptualization, Data curation, Formal analysis, Investigation, Visualization, Writing – original draft, Writing – review & editing. EA: Data curation, Investigation, Methodology, Validation, Writing – original draft. AM: Data curation, Formal analysis, Investigation, Methodology, Writing – review & editing. GR: Data curation, Investigation, Writing – review & editing. VU: Data curation, Investigation, Methodology, Writing – review & editing. AP: Data curation, Investigation, Methodology, Writing – review & editing. MN: Data curation, Investigation, Methodology, Validation, Writing – review & editing. CL: Formal analysis, Investigation, Validation, Visualization, Writing – review & editing. MBN: Data curation, Investigation, Methodology, Writing – review & editing. PF: Conceptualization, Supervision, Validation, Visualization, Writing – review & editing, Writing – original draft.
