# Supplementary material for: TMEM219 regulates the transcription factor expression and proliferation of beta cells
Source: Front Endocrinol (Lausanne). 2024 Jan 22;15:1306127. doi: 10.3389/fendo.2024.1306127 (PMC10839017; doi:10.3389/fendo.2024.1306127)
Supplement: Supplementary file 1 [file DataSheet_1.docx]

**Supplementary Materials**

**TMEM219 regulates the transcription factor expression and proliferation of beta cells**

Francesca D’Addio^1,2#^, Emma Assi^1#^, Anna Maestroni^1^, Giada Rossi^1^, Vera Usuelli^1^, Adriana Petrazzuolo^1^, Marta Nardini^1,4^, Cristian Loretelli^1^, Moufida Ben Nasr^1,3^,

Paolo Fiorina^1,2,3*^

^1^International Center for T1D, Pediatric Clinical Research Center Romeo ed Enrica Invernizzi, DIBIC, Università di Milano, Milan, Italy; ^2^Division of Endocrinology, ASST Fatebenefratelli-Sacco, Milan, Italy; ^3^Nephrology Division, Boston Children’s Hospital and Transplantation Research Center, Brigham and Women’s Hospital, Harvard Medical School, Boston, MA, USA; ^#^Co-first authors ^*^Corresponding author

**Supplementary Methods**

***Pancreatic islets and beta cell lines***

Human islets were obtained from 11 cadaveric organ donors (mean ± SEM: age: 49.3±1.4 years, and purity: 74±7%) as shown in Supplementary Table 1, whose pancreatic islets were not suitable for organ donation and cultured as already described (1-3) or were purchased from a commercial source (#35002-04, Celprogen, Torrance, CA) and cultured as previously described (2). The rat beta cell line INS-1 832/13 (SCC207, Sigma-Aldrich Merck) was cultured as already reported (4).

*Insulinoma study*

Plasma (n=10) and tissue samples (n=5) were collected from 10 patients (age 54.4±4.6 years, 4 males and 6 females) with insulinoma who had undergone surgery in the previous 12 months. Insulinoma was located within the pancreatic tail (size of 14.8± 2.2 mm) and graded as NET G1 at the histopathology analysis in all subjects. Duration of symptoms was 4.7±1.5 years (mean ± SEM). Patients had a mean ± SEM BMI of 29.2±1.2 kg/m^2^, systolic blood pressure (BP) of 131±5 and diastolic BP of 78.5±2 mmHg. Plasma IGFBP3 levels were assessed by ELISA kit (RAB0235, Sigma Aldrich/Millipore).

***Flow sorting***

Human pancreatic islets of healthy subjects were cultured with standard medium for 72 h and dissociated by trypsinization. In order to select TMEM219^+^ and TMEM219^-^ cell subsets, trypsinized cells were blocked with donkey serum and then stained with rabbit anti-human TMEM219, followed by donkey anti-rabbit AlexaFluor488 (ThermoFisher Scientific). Stained cells were flow-sorted using a BD FACSAria Fusion (BD Biosciences) and analyzed by qRT-PCR.

***Flow cytometry***

In order to confirm proliferation of insulin positive cells, human dissociated pancreatic islet were also fixed and permeabilized with Fixation and Permeabilization Solution Kit (554714, BD Biosciences, San Jose, CA) and stained with guinea pig anti-insulin antibody (1:200, Guinea Pig, ThermoFisher Scientific, PA1-26938) followed by PE anti-guinea pig (1:200, ThermoFisher Scientific, A-11073). CellTrace Violet (BV421, BD Biosciences) was used to detect proliferation. A BD FACS Celesta flow cytometry system (BD Biosciences) was used to run samples and analysis was conducted with Flowjo software (Version 10, Tree Star, Ashland, OR).

***qRT-PCR analysis***

RNA from purified human islets/endocrine progenitors/INS-1 was extracted using TRIzol Reagent (Invitrogen, Carlsbad, CA) and qRT-PCR analysis was performed using TaqMan assays (Life Technologies). Data were normalized for the expression of ACTB, and ∆∆Ct (fold change) were calculated. Primers used are as following:

| **Gene Symbol** | **Refseq Accession #** | **Band Size (bp)** | **Reference Position** |
| --- | --- | --- | --- |
| *Human* |  |  |  |
| INS | NM 000207.2 | 126 | 252 |
| TMEM219 | NM 001083613.1 | 60 | 726 |
| ALDH | NM 000689.4 | 61 | 1173 |
| PDX1 | NM_000209.3 | 73 | 517 |
| HNF6 | NM 004498.2 | 76 | 1231 |
| CASP8 | NM 001080124.1 | 124 | 648 |
| MKI67 | NM_001145966.1 | 66 | 765 |
| NKX6.1 | NM_006168.2 | 93 | 677 |
| SOX9 | NM_000346.3 | 102 | 1060 |
| NGN3 | NM_020999.3 | 127 | 1137 |
| ACTB | NM_001101 | 174 | 730 |
| *Rat* |  |  |  |
| INS | NM_019129.3 | 95 | 385 |
| MKI67 | NM_001271366.1 | 104 | 1609 |
| ACTB | NM_031144.3 | 91 | 881 |

***Embryonic stem cell (h-ESC) flow cytometry***

Cells at stages 0 (h-ESCs), 1, 2, 3, 4 and 5 of differentiation were detached with Versene (ThermoFisher Scientific), blocked with donkey serum, and then stained with rabbit anti-human TMEM219, followed by donkey anti-rabbit AlexaFluor488 (ThermoFisher Scientific). Flow cytometry was performed using a BD FACS Celesta flow cytometry system (BD Biosciences) and analyzed using Flowjo software (Version 6, Tree Star, Ashland, OR).

**Supplementary Table 1**. **Demographic and clinical characteristics of human pancreatic islets donors**.

|  | **Healthy subjects**  **(n=11)** |
| --- | --- |
| Age (years ± SEM) | 49.3±1.4 |
| Gender (M/F) | 7/4 |
| BMI (Kg/m^2^) | 24.5 ± 0.7 |
| HbA1c (%) | 5.4 ± 0.1 |
| HbA1c (mmol/mol) | 36.2 ± 1.9 |

**Abbreviations**: SEM, standard error of the means; M, male; F, female; BMI, body mass index; HbA1C, glycated hemoglobin.

**Supplementary Figure 1**. **TMEM219 is expressed in pancreatic beta cell precursors**.

**(A1-A3)**. Representative pictures of PDX1^+^TMEM219^+^, ALDH^+^TMEM219^+^ and INS^+^TMEM219^+^ immunofluorescence staining with DAPI included in purified human islets of healthy donors. Original magnification 40X, scale bar 25 μm. **(B1**-**B4, C1**-**C3)**. Anecdotical pictures of immunofluorescence staining of PDX1, ALDH and TMEM219 in positive and negative controls (Betalox-5, CaCo2, MCF7 and h-Podo cell lines), Original magnification 63X, scale bar 10 μm.

**Supplementary Figure 2**. ***TMEM219 is expressed in* in vitro-*derived endocrine progenitors***.

**(A)**. Bar graphs representing mRNA expression (mean) of *NGN3*, *PDX1*, *HNF6*, *NKX6.1*, *SOX9*, *INS* and *TMEM219* in differentiated stem cells at stages 0, 1, 2, 3, 4 and 5. Experiments were performed in triplicate (n=3). **(B)**. Bar graph showing TMEM219 protein expression (ELISA) assessed in stem cells at different stages of *in vitro* maturation, from stage 1 to stage 5 (endocrine progenitors), (n=3). **(C**-**D)**. Representative flow cytometry histogram and quantitative bar graph comparing TMEM219 expression in differentiated stem cells at stages 3, 4 and in endocrine progenitors (n=3). **(E)**. Quantification of TMEM219 protein by ELISA in cell fractions of a beta cell line cultured in the presence/absence of IGFBP3 (50 ng/ml 72 hours). Data are expressed as mean ± standard error of the mean (SEM) unless otherwise reported. mRNA expression was normalized to *ACTB*.

**Abbreviations**: qRT-PCR, quantitative real-time polymerase chain reaction; h-ESC, human embryonic stem cells; SEM, standard error of mean.

**Supplementary Figure 3**. **TMEM219 signaling in h-ESC-derived endocrine progenitors**.

**(A**, **B**, **C)**. Bar graph depicting CASP8 protein and mRNA expression and cell death measured by ELISA (fold change) at 72 h in endocrine progenitors cultured with IGFBP3 and in the presence or absence of ecto-TMEM219. **(D**, **E**, **F)**. Bar graphs comparing mRNA normalized expression of *HNF6, PDX1* and *MKI67* in differentiated endocrine progenitors (stage 5) cultured *in vitro* with/without IGFBP3 and in the presence or absence of ecto-TMEM219 for 72 h (n=3). **(G)**. TMEM219 protein quantification *in vitro*-differentiated endocrine progenitors cultured with/without miR-129-2 mimic and its negative control (scramble), (n=3). Experiments were run in duplicates. **(H**, **I)**. Bar graph representing *MKI67* and *Insulin* mRNA relative expression in endocrine progenitors cultured with/without miR-129-2 mimic and its negative control (scramble), (n=3). **(J)**. Bar graph showing quantification of proliferating islet Insulin positive cells analyzed at flow cytometry cultured with/without miR-129-2 mimic and its negative control (scramble), (n=3). Proliferation index has been calculated as ratio of CellTrace MFI day 0/MFI day 2.

All experiment were performed in duplicate. Data are expressed as mean ± standard error of the mean (SEM) unless otherwise reported. mRNA expression was normalized to *ACTB*.

**Abbreviations**: CASP8, Caspase 8; ALDH, aldehyde dehydrogenase; PDX1, pancreatic and duodenal homeobox 1; HNF6, Hepatocyte nuclear factor 6; ecto-TMEM219, newly generated recombinant protein based on TMEM219 extracellular portion.

**References**

1. Petrelli A, Carvello M, Vergani A, Lee KM, Tezza S, Du M, et al. IL-21 is an antitolerogenic cytokine of the late-phase alloimmune response. *Diabetes.* 2011;60(12):3223-34.

2. D'Addio F, Maestroni A, Assi E, Ben Nasr M, Amabile G, Usuelli V, et al. The IGFBP3/TMEM219 pathway regulates beta cell homeostasis. *Nat Commun.* 2022;13(1):684.

3. Fiorina P, Folli F, Bertuzzi F, Maffi P, Finzi G, Venturini M, et al. Long-term beneficial effect of islet transplantation on diabetic macro-/microangiopathy in type 1 diabetic kidney-transplanted patients. *Diabetes Care.* 2003;26(4):1129-36.

4. Gheibi S, Cataldo LR, Hamilton A, Huang M, Kalamajski S, Fex M, et al. Reduced Expression Level of Protein Phosphatase PPM1E Serves to Maintain Insulin Secretion in Type 2 Diabetes. *Diabetes.* 2023;72(4):455-66.
